# Being a Target for Glycation by Methylglyoxal Contributes to Therapeutic Efficacy of Injectable Collagen Hydrogels Post-Myocardial Infarction

**DOI:** 10.3390/gels12010018

**Published:** 2025-12-24

**Authors:** Xixi Guo, Ramis Ileri, Marc Ruel, Emilio I. Alarcon, Erik J. Suuronen

**Affiliations:** 1 BioEngineering and Therapeutic Solutions (BEaTS), Division of Cardiac Surgery, University of Ottawa Heart Institute, 40 Ruskin Street, Ottawa, ON K1Y4W7, Canada; xguo@ottawaheart.ca (X.G.); rileri@ottawaheart.ca (R.I.); mruel@ottawaheart.ca (M.R.); ealarcon@uottawa.ca (E.I.A.); 2 Department of Cellular & Molecular Medicine, University of Ottawa, 451 Smyth Road, Ottawa, ON K1H8M5, Canada; 3 Department of Biochemistry, Microbiology, and Immunology, University of Ottawa, 451 Smyth Road, Ottawa, ON K1H8M5, Canada

**Keywords:** myocardial infarction, methylglyoxal, collagen hydrogel, glycation, cardiac function

## Abstract

Despite the advances in medical therapies for treating myocardial infarction (MI), morbidity and mortality rates remain high. Following MI, increased methylglyoxal (MG) production leads to the accumulation of advanced glycation end-products (AGEs), which contribute to adverse remodeling and to the deterioration of cardiac function. We previously reported that an injectable collagen type I hydrogel improves the repair and function of mouse hearts post-MI. Notably, we observed that the injected hydrogel was a target for MG-AGE glycation, and that there were less MG-modified proteins in the myocardium. In this study, we further evaluated this protective mechanism by pre-glycating the hydrogels and assessing their therapeutic efficacy for treating MI. In vitro experiments showed that the viability of macrophages was reduced when cultured with the glycated hydrogel in the presence of MG. In vivo, female C57BL/6 mice were randomly assigned to receive intramyocardial injections of one of three treatments: phosphate-buffered saline, normal collagen hydrogel, or MG-glycated hydrogel. After 28 days, echocardiography was performed to evaluate cardiac function, and hearts were harvested for immunohistochemistry. Our results showed that the MG-glycated hydrogel had a reduced treatment effect (greater scar size, fewer wound-healing macrophages, less viable myocardium and decreased cardiac function) compared to mice that received the normal collagen hydrogel. In summary, this study demonstrates that the ability of the collagen hydrogel to act as a target for glycation and remove MG from the environment contributes to its therapeutic effect in treating the post-MI heart.

## 1. Introduction

Cardiovascular disease (CVD) is the major cause of non-communicable disease-related mortality worldwide, with an estimated 20.5 million deaths in 2021 [1]. The primary conventional treatments for myocardial infarction (MI; commonly known as a heart attack) are percutaneous coronary intervention (PCI) and coronary artery bypass grafting (CABG), both of which aim to revascularize the myocardium and reduce the mortality rate [2]. Despite the ability of PCI and CABG to improve patient outcomes, long-term mortality remains high, with a death rate above 20% over a 10-year period [3,4]. This necessitates the need to develop new strategies to limit tissue damage, reduce adverse cardiac remodeling and improve cardiac function in these patients.

The association of advanced glycation end products (AGEs) with aging and metabolic disorders has been well-established [5]. AGEs are a heterogeneous group of molecules that can modify the long-lived proteins of the extracellular matrix (ECM) through non-enzymatic glycation and they can interact with cells through the receptor for advanced glycation end-products (RAGE) [5]. Such AGE modifications can lead to cellular and tissue dysfunction and wound healing defects [6,7,8]. Methylglyoxal (MG), a highly reactive α-dicarbonyl compound derived mainly through glycolysis, is the primary precursor for AGE formation [9]. In healthy physiological conditions, MG is predominantly detoxified via the enzyme glyoxalase-1 (Glo1) and the glyoxalase system [10]. Post-MI, MG-AGE accumulation has been shown to play a role in adverse remodeling and functional deterioration of the heart [11]. Notably, reducing MG in the infarcted myocardium can limit adverse remodeling and improve cardiac function in a mouse MI model [12]. Furthermore, a clinical study observed that increased MG plasma levels in patients 24 h after MI was associated with lower cardiac function after 4 days [13]. Taken together, this evidence suggests MG as a promising therapeutic target for treating patients with MI.

Collagen-based hydrogel therapies have shown promise for treating MI by promoting cardiac repair and improving myocardial function [14,15,16]. For example, an injectable hydrogel using recombinant human collagen type I was shown to restore myocardial mechanical properties, reduce scar size, maintain remote wall thickness, and prevent heart enlargement, which led to improved myocardial contractility and function [15]. Subsequent studies revealed that this collagen-based hydrogel was able to sequester MG and reduce its levels in the surrounding myocardial tissue [17], which is consistent with the fact that collagen is a major target for MG glycation [18]. These findings suggest that the ability of the collagen hydrogel to act as a chemical “sponge” and soak up MG in the infarcted myocardium likely contributes significantly to its therapeutic effects. To further explore this mechanism, this study sought to compare the therapeutic efficacy of collagen hydrogels vs. MG-pre-glycated collagen hydrogels in a mouse model of MI.

## 2. Results and Discussion

### 2.1. Glycated Collagen Hydrogel Fails to Protect Macrophages from Methylglyoxal-Induced Cytotoxicity

**Figure 1:**
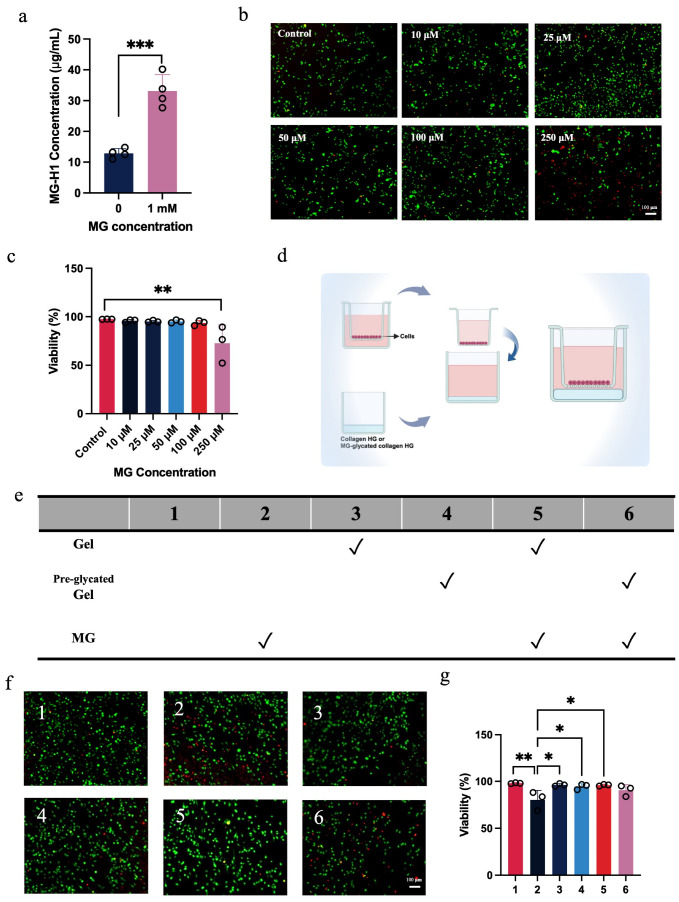
Glycated collagen hydrogel fails to protect the viability of MG-exposed macrophages. (**a**) MG-H1 concentration of collagen hydrogel treated with 0 or 1 mM of MG determined by an ELISA assay (*n* = 4; *** *p* < 0.001). (**b**) Representative images of macrophages stained using a Live/Dead assay (green = live, red = dead) after 24 h of culture with different concentrations of MG (scale bar = 100 μm). (**c**) Quantification of macrophage viability (*n* = 3; ** *p* < 0.01). (**d**) Schematic for the set-up of the wells and chambers for the cell experiments. (**e**) Experimental groups for the in vitro experiments. (**f**) Representative images of macrophages stained using a Live/Dead assay (green = live, red = dead) after 24 h of culture with collagen hydrogel or glycated collagen hydrogel in medium with or without MG (scale bar = 100 μm). (**g**) Quantification of macrophage viability for the conditions 1 through 6 shown in (**e**) (*n* = 3; * *p* < 0.05; ** *p* < 0.01).

We first confirmed that the collagen hydrogel used in this study was effectively glycated by MG. To this end, a collagen hydrogel was prepared by mixing 1 mM MG into the hydrogel solution prior to gelation. MG-derived hydroimidazolone (MG-H1) is the primary MG-AGE, which is formed by MG reacting non-enzymatically with arginine residues in proteins [10,19]. An ELISA assay was used to measure MG-H1 levels in both normal and MG-glycated hydrogels. An increase in MG-H1 was observed in the MG-treated collagen hydrogel compared to the untreated hydrogel (Figure 1a), demonstrating effective glycation of the hydrogel.

Following confirmation of the glycated status of the hydrogel, we then performed in vitro studies to demonstrate that the modified hydrogel had reduced protective effects on cells. We previously demonstrated that treatment with a collagen hydrogel promoted a greater number of pro-wound healing macrophages and reduced inflammation in the MI heart [15,20]. Since MG has been shown to promote inflammation and regulate macrophage behavior [21], we investigated the ability of the collagen hydrogel to protect macrophages from MG cytotoxicity. If the collagen hydrogel can protect macrophages by acting as a target for MG glycation, then pre-glycating the hydrogel should reduce its protective effect. To test this, macrophages were cultured in the presence of MG with normal and glycated collagen hydrogels after first determining the toxic concentration of MG and confirming that the collagen hydrogel could be effectively glycated by MG.

In healthy conditions, the physiological concentration of MG typically ranges from 1 to 4 µM, but its level increases in pathological conditions such as diabetes and post-MI [6,8,10]. To determine the toxic concentration of MG for macrophages, cells were cultured with MG at concentrations ranging from 10 µM to 250 µM (to mimic chronic pathological MG exposure in vivo) and viability was assessed using a Live/Dead assay. Results showed that 250 µM of MG significantly reduced macrophage viability (Figure 1b,c). Therefore, 250 µM of MG was selected as the toxic concentration for subsequent in vitro experiments.

To assess if pre-glycation compromised the protective effect of the collagen hydrogel, macrophages were cultured with either hydrogel type using standard and MG-containing media for 24 h (Figure 1d). Live/Dead assay results showed that neither collagen hydrogel nor glycated collagen hydrogel directly affected macrophage viability in standard media. However, upon exposure to MG, the collagen hydrogel protected cells from MG-induced cytotoxicity, while the glycated collagen hydrogel failed to confer protection (Figure 1f,g).

### 2.2. Glycated Collagen Hydrogel Fails to Improve Cardiac Function Post-MI

**Figure 2:**
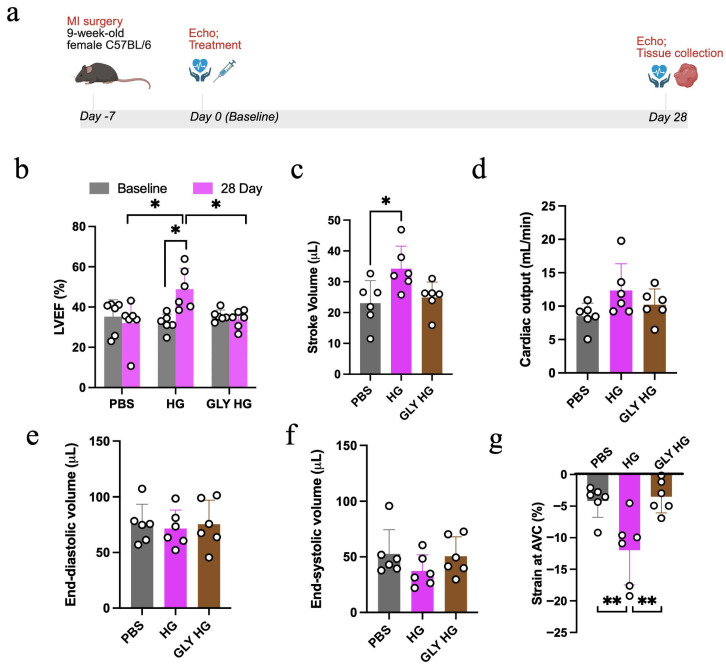
In vivo study timeline and cardiac function assessment of mice treated with PBS, collagen hydrogel (HG) or glycated collagen hydrogel (GLY HG). (**a**) Schematic of the study timeline. (**b**) Left ventricular ejection fraction (LVEF) at baseline (time of treatment delivery) and at 28 days post-treatment (*n* = 6; * *p* < 0.05). (**c**) Stroke volume (SV) (*n* = 6; * *p* < 0.05), (**d**) cardiac output, (**e**) end-diastolic volume (EDV) and (**f**) end-systolic volume (ESV) at 28 days post-treatment. (**g**) Strain force reached at the aortic valve closure (AVC) within the mid-anterior LV wall at 28 days post-treatment for the different experimental groups (*n* = 6; ** *p* < 0.01).

To evaluate the therapeutic efficacy of the normal and glycated collagen hydrogels, we used a mouse MI model. MI was surgically induced, and 1 week later, mice were randomly assigned to receive intramyocardial injections of phosphate-buffered saline (PBS; control), collagen hydrogel or glycated collagen hydrogel. After 28 days, there was no significant difference in the heart rate between mice treated with PBS (381 ± 21 bpm), collagen hydrogel (371 ± 11 bpm) or glycated collagen hydrogel (409 ± 11 bpm). Treatment with the collagen hydrogel led to a significant improvement in LVEF from baseline to 28 days post-treatment, whereas LVEF remained unchanged in the mice treated with the glycated collagen hydrogel or PBS (Figure 2b). Furthermore, LVEF at 28 days was significantly higher in hearts treated with collagen hydrogel compared to hearts treated with PBS or glycated collagen hydrogel (Figure 2b).

In terms of other cardiac function parameters, collagen hydrogel treatment resulted in an increase in stroke volume (SV) compared to PBS-treated hearts (Figure 2c), with a trend observed for an increase in cardiac output (*p* = 0.09). There were no differences in the end-diastolic volume (EDV) or the end-systolic volume (ESV) between the different treatments (Figure 2d–f). A significant improvement in the strain in the mid-anterior left ventricular wall at end systole was observed at 28 days after collagen hydrogel injection (Figure 2g), but not after PBS or glycated collagen hydrogel treatment. This strain analysis provides evidence that the collagen hydrogel injection improves cardiac contractility compared to PBS- and glycated collagen hydrogel-treated animals.

### 2.3. Greater Scar Formation and Less Preservation of Cardiac Muscle After Glycated Collagen Hydrogel Treatment

**Figure 3:**
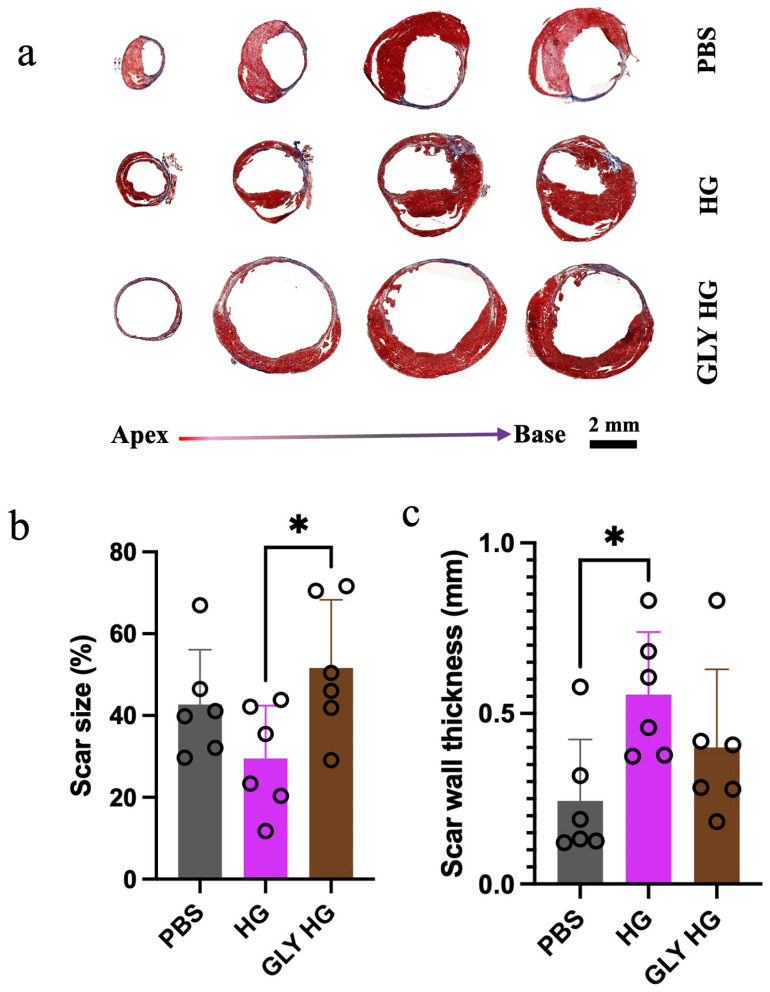
Histological assessment of scar size and scar wall thickness post-treatment. (**a**) Representative Masson’s trichrome-stained sections of hearts harvested at 28 days post-treatment (scale bar = 2 mm). (**b**) Scar size (% of LV) measured at 28 days post-treatment from Masson’s trichrome stained tissue sections (*n* = 6; * *p* < 0.05). (**c**) Myocardial wall thickness of the scar measured from Masson’s trichrome-stained tissue sections at 28 days post treatment (*n* = 6; * *p* < 0.05).

**Figure 4:**
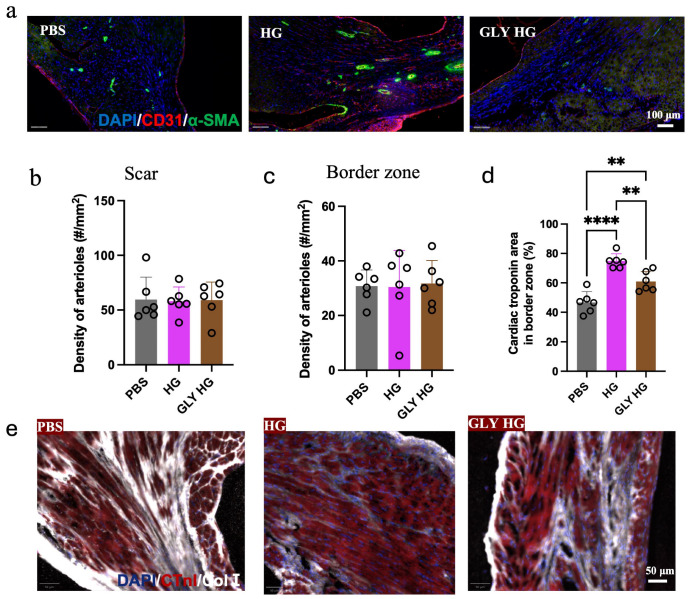
Immunohistochemistry assessment of vascular density and cardiac muscle after treatment. (**a**) Representative images of heart tissue sections stained with CD31 (red), α-smooth muscle actin (green) and DAPI (blue) for the different treatment groups (scale bar = 100 μm). (**b**,**c**) Quantification of arteriole density (number per mm^2^ (#/mm^2^)) in the myocardial scar and border zone after treatment with PBS, collagen hydrogel (HG) and glycated collagen hydrogel (GLY HG; *n* = 6). (**d**) Quantification of percentage of cardiac troponin I^+^ area in the border zone for the different groups (*n* = 6; ** *p* < 0.01; **** *p* < 0.0001). (**e**) Representative images of heart tissue sections from the border zone stained with DAPI (blue), cardiac troponin I (red) and collagen type I (white) for the different treatment groups (scale bar = 50 μm).

Masson’s trichrome staining at 28 days revealed a reduction in scar size in the hearts treated with the collagen hydrogel compared to those treated with the glycated collagen hydrogel (Figure 3a,b). Also, the scar wall thickness was increased in collagen hydrogel-treated hearts compared to the PBS treatment, which was not observed for hearts that received the glycated collagen hydrogel (Figure 3c). Immunohistochemistry was also performed to assess vascular density in the scar and border zone at 28 days post-treatment. No differences in arteriole density were observed in the scar area or border zone between the different treatment groups (Figure 4a–c). Analysis of the heart sections also included staining for cardiac troponin I to assess the level of surviving cardiomyocytes. Quantification of the percentage of cardiac troponin I^+^ area in the border zone revealed that treatment with the collagen hydrogel resulted in the greatest preservation of cardiac muscle area. While the glycated collagen hydrogel treated hearts also had greater cardiac troponin I^+^ area compared to the PBS group, it was significantly less than that observed for the collagen hydrogel treatment.

### 2.4. Collagen Hydrogel Enhances M2 Macrophage Recruitment in the Scar

**Figure 5:**
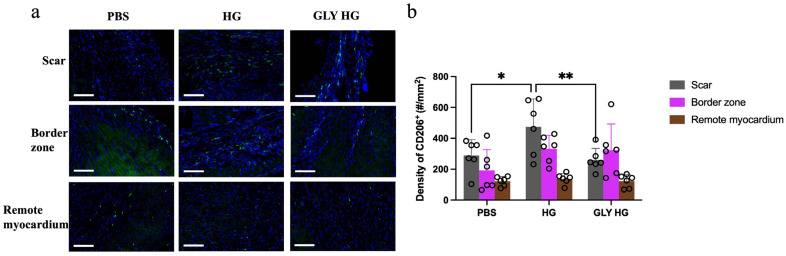
Immunohistochemistry assessment of wound healing macrophage density after treatment. (**a**) Representative images of heart tissue sections stained with CD206 (green) and DAPI (blue) for the different treatment groups (scale bar = 100 μm). (**b**) Quantification of the number of CD206^+^ macrophages (number per mm^2^ (#/mm^2^)) in the scar, border zone and remote myocardium of MI hearts treated with PBS, collagen hydrogel (HG) and glycated collagen hydrogel (GLY HG; *n* = 6; * *p* < 0.05; ** *p* < 0.01).

Massive cell death that occurs after MI activates inflammation and macrophage recruitment in the myocardium. A greater ratio of pro-wound healing M2 macrophages to the pro-inflammatory M1 macrophages has been shown to mitigate chronic inflammation and enhance cardiac repair post-MI [22,23]. In our study, 28 days after collagen hydrogel treatment, the number of CD206^+^ M2 macrophages in the scar region was approximately two-fold higher compared to the PBS and glycated collagen hydrogel groups (Figure 5a,b), but no differences were observed between groups in the border zone or remote myocardium.

### 2.5. Discussion

Collagen-based hydrogels have been widely explored for treating the infarcted heart. Depending on the formulation used, such therapies have been shown to provide a variety of reparative benefits including increased vascularization, reduced inflammation and fibrosis, and enhanced cell survival, leading to reduced scar size, less adverse remodeling and improved cardiac function [24,25,26,27]. This suggests that collagen hydrogels can activate numerous repair pathways, for which many underlying mechanisms remain to be elucidated. In this study, we investigated a potential mechanism whereby the collagen hydrogel may act as a “sponge” to remove MG (toxic dicarbonyl) that accumulates in the heart following MI.

MG-derived AGEs contribute to cell and tissue dysfunction in many diseases, including cardiovascular disease and myocardial infarction [6,11]. These products are formed by the non-enzymatic crosslinking of MG to lysine and arginine residues in proteins, with high reactivity observed with the arginines of collagen in the ECM [10,19]. The high affinity of MG for collagen led to the hypothesis that collagen-based hydrogels may confer therapeutic benefits in cardiac repair through their ability to act as a target for MG glycation. Notably, we previously reported that our collagen hydrogel was able to sequester MG and reduce its levels in the surrounding myocardial tissue [17]. To evaluate this observation more mechanistically, in this study we treated MI mouse hearts with collagen hydrogels that had been pre-treated with MG. By blocking their ability to “trap” MG, this allowed us to directly assess the importance of this mechanism in their therapeutic effect. Our results demonstrated that the MG-trapping function of the collagen hydrogel is critical for its ability to reduce scar size and adverse remodeling, to promote wound healing macrophages, to preserve viable myocardium and to improve cardiac contractility and function of the infarcted heart.

The glycated collagen hydrogel was synthesized by introducing MG into a sealed mixing system at a final concentration of 1 mM. The injectability of the hydrogel solution was maintained after the addition of MG and the glycated status of the formed hydrogel was confirmed. A limitation of this study is that the potential for the added MG to interfere with the crosslinking process and hydrogel formation was not assessed. Visual inspection confirmed that both hydrogels were structurally intact and capable of maintaining their shape in vitro, indicating that the addition of MG did not lead to a failure of the crosslinking process. However, more comprehensive analyses, such as rheology, are needed to better compare the physical properties of the 2 hydrogels. Despite this, our previous work showed that most of the collagen hydrogel does not persist in the heart beyond 3–7 days after its delivery [12,15]; suggesting that the effect of the hydrogel’s mechanical properties on its therapeutic efficacy is likely minimal compared to its MG-trapping capacity.

Previous studies have demonstrated the ability of our collagen hydrogels to modulate the function of many cell types [15,20]. For example, the collagen hydrogel was shown to mediate inflammatory cell function and inflammation post-MI [15]. Based on this and for the purpose of confirming that glycation reduces the ability of the collagen gel to protect cells from MG, we used macrophages for the in vitro studies herein. The glycated hydrogel exhibited no toxicity towards macrophages in culture. While the normal collagen hydrogel prevented macrophage death in the presence of a toxic level of MG in the culture medium, the glycated collagen hydrogel failed to protect macrophages from MG-induced cytotoxicity. This observation suggests that glycation of arginine residues within the collagen hydrogel limits its capacity to sequester additional MG from the medium, thus allowing MG to target and negatively affect the macrophages.

In the mouse MI model, treatment with the glycated collagen hydrogel failed to confer the same benefits to cardiac repair that was seen in mice that received the normal collagen hydrogel. While cardiac function was improved with collagen hydrogel injection, this was not observed when hearts were treated with glycated collagen hydrogel. Unlike some matrix therapies that rely on passive mechanical restriction to limit post-infarct remodeling (ventricular dilation), the preservation of LVEF in the collagen hydrogel group was achieved without a change in end diastolic volume. This suggests that the therapeutic benefit is driven primarily by improved LV contractility rather than by structural reinforcement. This is supported by the improvement in global longitudinal strain and the preservation of viable cardiomyocytes observed in the collagen hydrogel treated mice. Furthermore, the scar size was greater and the area of viable cardiac muscle in the border zone was less in the mouse hearts treated with the glycated hydrogel compared to the normal hydrogel, suggesting that glycation reduced the ability of the hydrogel to limit adverse remodeling of the myocardium. This highlights that the collagen hydrogel being a target for MG glycation plays an important role in its therapeutic efficacy. Notably, the hearts treated with the collagen hydrogel exhibited a greater number of M2 wound healing macrophages in the scar region, which was not observed for the glycated hydrogel treatment group. Although the hydrogel was injected into the border zone, labeling and imaging methods have shown that our collagen hydrogels spread across the infarct and border zone areas following their injection [12,15]. This allows for the collagen hydrogel to exert its effects directly in both the scar and border zone. Modulating macrophages to a wound healing phenotype can limit adverse remodeling in the MI heart [28], while the presence of MG has been shown to promote inflammatory cytokine production and disrupt the balance between inflammatory (M1) and wound healing (M2) macrophages [29,30]. Thus, the removal of MG from the myocardium by the collagen hydrogel to promote a wound healing environment rather than an inflammatory one may be one possible mechanism for the observed benefits of collagen hydrogel treatment.

The ability to act as a target for glycation and remove toxic MG from the environment is not expected to be exclusive to biomaterials made from collagen. Although collagen is a primary target for MG, any material with amino acid residues that can be glycated are also likely to provide a similar MG removal function leading to some level of cardioprotection. A future consideration in the application of such MG-trapping materials would be the timing of delivery. In the present study, the collagen hydrogel was injected at 7 days post-MI during the proliferative phase of infarct healing, which is the same time-point as our previous work looking at MG in collagen hydrogel-treated hearts. In the previous work, MG-H1 modification of the injected collagen hydrogel was shown, while reduced MG-H1 levels were observed in the myocardial ECM and in cardiomyocytes [17], suggesting that the hydrogel was able to scavenge MG being produced at 7 days post-MI and protect the myocardium from its effects. However, we have also shown that MG begins to accumulate acutely post-MI (as early as 6 h after infarction) [11], and our collagen hydrogel is more effective at protecting the myocardial environment and improving cardiac function when applied early (at 3 h post-MI) [20]. This suggests that delivering the collagen hydrogel soon after infarction may better protect the myocardial environment and improve cardiac function by removing MG early and limiting its damaging effects, which constitutes a mechanism for future investigation. An attractive feature of injectable hydrogel therapies is that they can be delivered in a minimally invasive manner [31]. Given this, future research could also consider comparing the therapeutic efficacy of MG-trapping materials delivered in multiple injections at different time-points versus a single time-point injection.

## 3. Conclusions

In summary, our findings demonstrate that the ability of the collagen hydrogel to act as a target for MG glycation contributes significantly to its efficacy as a therapy for treating MI. When its MG-tapping capacity was blocked, it failed to prevent adverse cardiac remodeling, promote wound healing macrophages, protect myocardial viability and enhance cardiac function. This further highlights MG as a potential target for post-MI treatment, suggesting the design of specific inhibitors or the use of existing biomaterials or pharmacological agents that target MG to improve outcomes in patients post-MI.

## 4. Materials and Methods

### 4.1. Preparation of the Collagen Hydrogel

The collagen hydrogel preparation was conducted following our previously described protocol [20]. Briefly, type I rat-tail collagen (3–4 mg/mL) was obtained from Corning (CLS354236; Bedford, MA, USA). Chondroitin sulfate (CS; Fujifilm Wako Pure Chemical Corporation, Osaka, Japan), N-ethyl-N-(3-dimethylaminopropyl) carbodiimide (EDC) from MilliporeSigma (Oakville, ON, Canada), and N-hydroxysuccinimide (NHS) from MilliporeSigma (Oakville, ON, Canada) were added to the collagen solution to a final mass ratio of 1:4:0.5:0.3 for collagen–CS:NHS:EDC. The hydrogel mixture was prepared on ice using an enclosed syringe system that ensured homogeneous mixing and prevented bubble formation. After mixing thoroughly, 1.0 N NaOH was used to adjust the solution to a pH of 7.4. The glycated collagen hydrogel with the same collagen–CS:NHS:EDC ratio was prepared using the same procedure, with the addition of MG at a final concentration of 1 mM. This concentration of MG was used since it has been shown to glycate collagen in vitro at a level comparable to that seen in the ventricles of rats with diabetic cardiomyopathy [32].

### 4.2. ELISA Analysis

An ELISA kit (Cell Biolabs Inc., Burlington, ON, Canada, STA-811) was used to quantify MG-H1 levels and confirm glycation of the collagen hydrogel by MG treatment. Hydrogels were prepared as described above, rinsed for 5 min in PBS, and approximately 45 mg of each sample was collected, minced and put into a tube. RIPA buffer containing a protease inhibitor was added, followed by sonication and centrifugation at 13,000 rpm for 15 min at 4 °C. The supernatant was collected and analyzed using the ELISA kit according to the manufacturer’s protocol.

### 4.3. Cell Culture and Viability Assay

Bone marrow-derived RAW macrophages were cultured in Macrophage-SFM (1×) (Thermo Fisher Scientific, Burlington, ON, Canada, 12065074) supplemented with 10% fetal bovine serum at 37 °C in a humified incubator.

To determine the concentration of MG that resulted in a loss in macrophage viability, cells were cultured in an incubator for 24 h with MG at concentrations ranging from 10 to 250 µM. Following incubation, cell viability was assessed using the Live/Dead assay kit (Calcein AM/EthD-1; Invitrogen™, Burlington, ON, Canada, L3224).

To assess the protective effects of hydrogels on cell viability, six experimental groups were tested. Macrophages were cultured with no hydrogel, collagen hydrogel, or glycated collagen hydrogel in media with or without 250 µM of MG in a Transwell plate (Corning, 3470). Cells were seeded in the top chamber, while the hydrogel or glycated collagen hydrogel was placed in the bottom chamber. After 24 h, the medium was removed and cell viability was assessed using the Live/Dead kit (Invitrogen™ L3224). Imaging was performed using a Zeiss microscope, and the number of live and dead cells was quantified using ImageJ software (Version: 2.14.0/1.54f). The viability (% live cells) was calculated as follows:

$$\mathrm{Viability}=\frac{{number}_{Live\;cells}}{{{number}_{live\;cells}+number}_{dead\;cells}}\;\times \;100\%.$$

### 4.4. Myocardial Infarction Model

All procedures were approved by the University of Ottawa Animal Care Committee (protocol # HIe-3402; approval date: 21 October 2022) and conducted in accordance with the National Institutes of Health (NIH) Guide for the Care and Use of Laboratory Animals.

MI was induced in 9-week-old female C57BL/6 mice, and treatment delivery followed our established protocol [15]. Briefly, mice were anesthetized with 2% isoflurane, intubated, and then underwent a fourth intercostal thoracotomy procedure to expose the heart. The left anterior descending coronary artery was ligated just below its emergence from the left atrium, leading to a large MI involving the anterolateral, posterior, and apical regions of the heart. The observation of myocardial blanching confirmed successful infarction. Short-acting buprenorphine was administered at least one hour before surgery, and long-acting meloxicam was given subcutaneously immediately before surgery for perioperative analgesia.

One-week after MI (baseline), mice were randomly assigned to receive treatment with PBS (control), collagen hydrogel, or glycated collagen hydrogel via five intramyocardial injections (10 µL per site, 50 µL total) using a 27-G needle under ultrasound-guided closed-chest procedure. After securing the syringe with a micromanipulator (VisualSonics, Toronto, ON, Canada), both the needle and RMV scanhead probe were aligned along the heart’s long axis. The needle tip was positioned in the myocardium under ultrasound guidance before injection. Mice were euthanized by terminal anesthesia at 4 weeks post-treatment, and the hearts were collected for histological analysis.

### 4.5. Echocardiography

A Vevo770 system in B mode with a 707B series real-time micro visualization scanhead probe (VisualSonics) was used to perform transthoracic echocardiography in long-axis views. Imaging was conducted at 7 days post-MI and at 28 days post-injection to evaluate the following cardiac functional parameters: left ventricular ejection fraction, fractional area change, end-systolic volume, end-diastolic volume, stroke volume, and cardiac output. Strain analysis was conducted using transthoracic echocardiography with a Vevo3100 system in B mode, utilizing an MX400 series real-time micro visualization scanhead probe (VisualSonics). Imaging was performed at 7 days post-MI and at 28 days post-injection to assess longitudinal endocardial strain at aortic valve closure (end systole). The VevoStrain application in Vevo LAB 3.1.1 software (VisualSonics) was used to analyze strain in segment 5 (the anterior mid-segment), corresponding to the border zone targeted for injection.

### 4.6. Histology and Immunohistochemistry

At 28 days post-injection, mice were euthanized by terminal anesthesia. Hearts were harvested, perfused with PBS, embedded in OCT, and snap-frozen in liquid nitrogen. Sections (10 µm thick) were cut using a cryostat, with the collection of 8 sections per heart starting at 300 µm from the apex.

Scar size was assessed by staining tissue sections with Masson’s trichrome after fixation in 4% paraformaldehyde (PFA) for one hour. Images were captured using an Olympus BX50 microscope with a 2× objective. Sections of myocardial tissue at approximately 450 µm were analyzed to determine scar wall thickness and scar size, which were calculated using the mid-line arc method in ImageJ software (Version: 2.14.0/1.54f).

For immunohistochemical analysis, tissue sections were fixed in acetone for 20 min, permeabilized with 0.1% Triton for 10 min, and blocked with 10% serum for one hour at room temperature. Primary antibodies were applied overnight at 4 °C in 10% serum, followed by secondary antibody treatment for one hour at room temperature. Slides were mounted using fluorescent mounting medium (Dako, Oakville, ON, Canada). To assess arteriole numbers, primary antibodies targeting PECAM-1 (CD31; Santa Cruz, Dallas, TX, USA, 101454, 1:50) and α-SMA (Abcam, Cambridge, UK, 5694, 1:200) were used. with the secondary antibodies AF594 anti-rat (Life Technologies, Burlington, ON, Canada, A11008, 1:500) and AF488 anti-rabbit (Life Technologies A11007, 1:500) were then applied to detect PECAM-1 and α-SMA, respectively. M2 macrophages were identified using an AF488-conjugated anti-CD206 antibody (BioLegend, San Diego, CA, USA, 141710, 1:50). Heart sections were also stained with primary antibodies targeting Collagen type I (Abcam 316222, 1:500) and cardiac troponin I (Abcam 188877, 1:200), which were detected with AF647 anti-rabbit (Life Technologies A31573, 1:1000) and AF568 anti-goat (Life Technologies A11057, 1:200) secondary antibodies, respectively. Fluorescent images were obtained using a Leica Aperio Versa 8 microscopy (Concord, ON, Canada) with a 20× objective. Sections at around 450 µm from the apex were analyzed using QuPath. For each section, the analysis was performed using 2–4 images taken within the border zone, infarct, and remote regions. All histology image analysis was performed and validated independently by two investigators.

### 4.7. Statistical Analysis

Statistical analysis was performed using Prism 9. All data are presented as the mean ± SEM. For the comparison of in vivo data between treatments, an ANOVA was used followed by Holm’s correction for multiple comparisons. In vitro, the data was analyzed either by a Student’s *t*-test or by one-way ANOVA with a Holm’s correction for multiple comparisons.

## Data Availability

All data generated or analyzed in this study are included in the manuscript. Other raw and processed data that support the findings of this study are available upon request.
